# Quantification of glycated hemoglobin and glucose in vivo using Raman spectroscopy and artificial neural networks

**DOI:** 10.1007/s10103-022-03633-w

**Published:** 2022-09-05

**Authors:** Naara González-Viveros, Jorge Castro-Ramos, Pilar Gómez-Gil, Hector Humberto Cerecedo-Núñez, Francisco Gutiérrez-Delgado, Enrique Torres-Rasgado, Ricardo Pérez-Fuentes, Jose L. Flores-Guerrero

**Affiliations:** 1 Optics Coordination, National Institute of Astrophysics, Optics and Electronics (INAOE), 72840 Puebla, Mexico; 2 Computer Science Coordination, National Institute of Astrophysics, Optics and Electronics (INAOE), 72840 Puebla, Mexico; 3 Faculty of Physics, University of Veracruz (UV), 91090 Veracruz, Mexico; 4 Center for Cancer Studies and Prevention (CEPREC), 29038 Tuxtla Gutiérrez, Chiapas Mexico; 5 Faculty of Medicine, Meritorious Autonomous University of Puebla (BUAP), 72589 Puebla, Mexico; 6 Department of Chronic Disease Physiopathology, East Center of Biomedical Research, Mexican Social Security Institute (CIBIOR), 74360 Puebla, México; 7 MRC Unit for Lifelong Health and Ageing, Institute of Cardiovascular Science, University College London, London, WC1E 7HB UK

**Keywords:** Raman spectroscopy, Artificial neural networks, Glucose, Diabetes, HbA1c, In vivo measurements

## Abstract

Undiagnosed type 2 diabetes (T2D) remains a major public health concern. The global estimation of undiagnosed diabetes is about 46%, being this situation more critical in developing countries. Therefore, we proposed a non-invasive method to quantify glycated hemoglobin (HbA1c) and glucose in vivo. We developed a technique based on Raman spectroscopy, RReliefF as a feature selection method, and regression based on feed-forward artificial neural networks (FFNN). The spectra were obtained from the forearm, wrist, and index finger of 46 individuals. The use of FFNN allowed us to achieve an error in the predictive model of 0.69% for HbA1c and 30.12 mg/dL for glucose. Patients were classified according to HbA1c values into three categories: healthy, prediabetes, and T2D. The proposed method obtained a specificity and sensitivity of 87.50% and 80.77%, respectively. This work demonstrates the benefit of using artificial neural networks and feature selection techniques to enhance Raman spectra processing to determine glycated hemoglobin and glucose in patients with undiagnosed T2D.

**Supplementary Information:**

The online version contains supplementary material available at 10.1007/s10103-022-03633-w.

## Introduction

Non-communicable diseases represent the leading cause of death worldwide. Due to its global rise in incidence and prevalence, type 2 diabetes (T2D) is considered among the top deadliest diseases, accounting for 1.6 million deaths annually [1]. According to the last report of the Global Burden of Diseases, Injuries, and Risk Factors Study, high plasma glucose belongs to the top three risk factors with the largest increase in the world during the last decade [2]. T2D itself had been considered the greatest pandemic in human history [3].

According to the International Diabetes Federation, the latest global diabetes prevalence (2019) is estimated to be 9.3%, accounting for 463 million people [4]. Nevertheless, it has been argued that such figures underestimate the real number of diabetes prevalence, by at least 25% [3]. Importantly, the underdiagnosis of T2D in low- and middle-income countries, where the resources to perform a T2D screening are limited, could be as high as 46% [5]. In order to cope with this public health problem, non-invasive techniques to determine the quantity of glucose and glycated hemoglobin (HbA1c) have been proposed recently, such as NIR spectroscopy, Raman spectroscopy, surface-enhanced Raman spectroscopy, and mid-infrared spectroscopy, among others [6–10].

Raman spectroscopy is an optical technique commonly used [9, 11–18]; its instrumentality relies on the interaction of electromagnetic radiation with matter, including biomolecules such as keratin, lipids, myoglobin hemoglobin, or glucose [19, 20]. As a result of this interaction, part of the incident light is scattered, and in most cases, the wavelength of the scattered photons remains constant; this is called Rayleigh scattering. However, a small part of the light is scattered at a different wavelengths concerning the incident wavelength due to the gained or lost energy after the interaction, which is called Raman scattering [21, 22]. As a result, a specific spectral signature of the analyzed molecule is obtained [23].

Raman spectral signature potentially identifies metabolites of clinical importance for T2D diagnostic [24, 25]. The gold standard is the HbA1c test, in addition to constant measurements of glucose in diagnosed patients with T2D [26]. Nevertheless, Raman spectroscopy has several types of signal noise, such as shot noise, fluorescence, readout noise, external source noises, and instrumentation-derived noise [27–30]. In order to reduce these signal noises, many techniques have been developed, for instance, Savitzky-Golay filter [31], wavelet transformation [32, 33], polynomial curve fit [34], baseline correction [35], empirical mode decomposition [36], the Vancouver Raman algorithm [36], and the Zernike polynomial fitting [37], among others.

Furthermore, artificial neural networks (ANN) have been proposed as suitable techniques for Raman spectra analysis [17, 38, 39]. There are different ANN architectures; for instance, the feed-forward neural network (FFNN) comprehends different neuron layers, on which the output from the neuron in the level *k* is connected to the input neuron in the level *k* + 1. The output of the network corresponds to the values of the neurons in the output layers [40]. The FFNN has been used both as a classification method and as a function approximation on Raman spectra analyses [41].

In vivo studies have explored the potential use of Raman spectroscopy for the quantification of T2D diagnosis biomarkers. For instance, the classification of 86 individuals as free from T2D, controlled T2D, and non-controlled T2D has been reported. In those analyses, the information obtained by Raman spectroscopy was analyzed using principal component analysis and support vector machines (SVM), showing > 90% of specificity and sensibility [16]. Furthermore, the use of ANN and SVM to discriminate between normoglycemia and hyperglycemia through the Raman spectra has been reported in a different population (eleven individuals), achieving 88.9 to 90.9% of specificity and sensibility [17].

In vivo quantification of circulating plasma glucose concentrations using Raman spectroscopy directly over the skin of the individuals, using the fingertip, has shown promising results. The calculated concentrations using linear regression were reported to be highly correlated with capillary glucose measurement, getting a correlation coefficient of 0.80 (*p* < 0.0001) in 49 individuals [42]. Raman spectroscopy readings from the forearms analyzed with partial least-squares (PLS) regression have also shown promising results (mean absolute error (MAE) 7.8%, (*N* = 17)) [43]. Furthermore, PLS and Raman spectroscopy were used to predict glucose concentration in the forearm of 111 individuals obtaining a correlation coefficient of 0.83 in independent Raman predictions for the full cohort [44]. In addition, critical-depth Raman spectroscopy and PLS were used to quantify circulating glucose in 35 individuals for a period of 60 days, obtaining a mean average relative difference (MARD) of 25.8% with 93% of predictions in the areas A and B of the Clarke error grid, in the independent validations [45]. Recently, it has been reported an improvement of the in vivo quantification of glucose with Raman spectroscopy, in which linear regression and PLS were applied to analyze the Raman spectra of pigs’ ears. The Raman readings showed almost perfect agreement with the gold standard, 0.94 correlation coefficient in intra-subject analyses [46].

Even though Raman spectroscopy and machine learning methods have been used for T2D-related biomarkers’ quantification, the in vivo quantification of glucose needs to be improved. Moreover, the in vivo quantification without blood extraction of HbA1c remains unexplored; being this the gold standard for diabetes detection, finding new methods for HbA1c quantification is relevant due to that HbA1c is not only a biomarker to evaluate the glucose control, but also a diagnostic one [47]. Therefore, the present study investigates whether Raman spectroscopy coupled with feature selection methods and FFNN is suitable for the non-invasive quantification of HbA1c and glucose in people with and without T2D diagnosis.

## Methods

A cross-sectional sectional study including 46 volunteer participants (16 men and 30 women from 27 to 87 years old) was conducted to perform the measurements of glucose and HbA1c, both with Raman spectroscopy and traditional methods. Blood samples were taken between 8:00 and 10:00 after an overnight fast and 15 min of rest prior to sample collection. Plasma samples were prepared by centrifugation at 4 °C. HbA1c and glucose are tested by boronated affinity method and glucose oxidase, respectively [48, 49]. Subsequently, Raman measurements were made in three different body parts: forearm, wrist, and index finger. Participants with full data available were included in the present analysis.

### In vivo measurements of glucose and HbA1c

The values of HbA1c varied from 5.2 to 14% and glucose values from 56 to 400 mg/dL. According to the HbA1c cut-off values for T2D (> 6.5%) and prediabetes diagnosis (5.7–6.4%) [50], 32 participants were T2D, ten were prediabetes, and four were healthy. Also, lyophilized glucose and lyophilized human HbA1c were acquired from Sigma–Aldrich Corporation, St Louis, MO, USA (ID product: G8270 and IRMMIFCC466, respectively) for their characterization.

The Raman measurement setup was composed of a Raman spectrometer QE65000 from Oceans Optics® with a resolution of 0.14–7.7 nm FWHM and a Raman probe InPhotonics® RIP-RPS-785 with 60 mW of power by optical decoupling [38]. The environmental conditions were humidity 63.09 ± 5.25% and temperature 19.76 ± 1.02 °C. Each measure was taken using 30 s of integration time, and nine measurements per body part and volunteer were taken. The laser power and integration time were calculated according to the American National Standard for safe use of lasers (ANSI Z136.1–2007) [51].

Given the fact that external lighting could be a source of noise for the Raman spectra [52], the measurements were performed in a room without light, and we implemented a cover on the Raman tip, which considers the focal length of 7 mm. This cover,  allowed us to block the external light from  the analyzed region. However, it was important that the volunteer did not move, since otherwise, the measurements may have alterations. The protocol for the present study was approved by the local ethics committee (approval number: CEPREC 08–001). All participants included in the presented analysis provided written informed consent to participate, and all study procedures were conducted according to the Declaration of Helsinki [53]. The present study follows the Reporting Diagnostic Accuracy Studies STARD 2015 EQUATOR (Supplementary Table S6).

### Spectral data analysis

Selecting the best representation of the Raman spectra is an important part of the spectral analysis in order to improve the results. Several methods have been used in Raman spectroscopy to obtain the best spectral representation, such as PCA [54], colony optimization [55], genetic algorithms [37], and support vector machine-recursive feature elimination [56], among others. In this work, self-organizing maps (SOM) network and RReliefF were proposed to obtain the best data representation.

The SOM network and RReliefF performance were compared with other feature selection methods such as correlation feature selection (CFS), wrapper method, and PCA. We found that the SOM network combined with RReliefF presented the minor RMSE-CV, consequently a better performance; this comparison is shown in the supplementary material (Tables S1–S4). SOM is an FFNN architecture trained with an unsupervised learning algorithm [57]. SOM network aims to find significant patterns or features in the input data and establishes a correspondence between this data and two-dimensional space, being possible to discover the regularities present in the data and extract features or group patterns according to their similarity [58].

ReliefF is an algorithm proposed by Kira and Rendell in 1992 [59]. The main idea is to estimate the quality of attributes according to how well their values distinguish between instances close to each other. In the case of ReliefF for regression problems (RReliefF), predictors that give different values to neighbors with the same response values are penalized, and predictors that provide different values to neighbors with different response values are rewarded. Predictor weights were listed by attribute importance, which facilitates attribute selection.

In this work, the spectral data were filtered to reduce fluorescence and shot noise using Zernike polynomial fitting combined with genetic algorithms and Whitaker filter, respectively [37]. Besides, RReliefF and SOM were implemented to obtain a better representation of data within a spectral interval from 200 to 1800 cm^−1^ (788 features). The parameters of RReliefF, such as k-nearest neighbors and the number of features, were varied using a search based on the FFNN performance. The features were varied as 50, 100, 150, 200, and 512 features and k-nearest neighbors from 5 to 40.

We used “neural net fitting” and “neural pattern recognition” from the toolbox “deep learning” by MATLAB 2019b to design the ANN. Its structure conforms by two layers (one hidden layer) with hyperbolic tangent sigmoid activation functions and Levenberg–Marquardt and scaled conjugate gradient as training algorithms. To compute the FFNN weights in regression and classification, the number of neurons in the hidden layer varied from 5 to 20 to determine the best combination based on the network performance. Besides, the “regression learner” toolbox from MATLAB 2019b was used to compare the results obtained from ANN to SVM and linear regression. Also, we implemented iPLS [60] in which the spectra were divided into ten subintervals from 200 to 1800 cm^−1^. In each interval, PLS and ANN were computed to compare both methods.

For classification, two spectral intervals (200–1800 cm^−1^ and 600–1600 cm^−1^) were used, and three classes were generated according to the HbA1c values (T2D, prediabetes, and healthy). Data were randomly divided into three sets (training 70%, validation 15%, and testing 15%). Finally, the network was executed 20 times in order to determine the total FFNN performance by using accuracy, specificity, and sensitivity [61].

For regression, the Raman spectral interval between 200 and 1800 cm^−1^ was used. The values to be approximated varied from 56 to 400 mg/dL for glucose and 5.2 to 14% for HbA1c (values obtained from laboratory measurements). Data were randomly divided into three folds (276 spectra for training and 138 for testing per fold), and cross-validation was done in order to prevent overfitting [62–64]. The metric used to measure the FFNN performance in the regression was the root mean square error in cross-validation (RMSE-CV) and the standard deviation (SD) between folds [65].

The clinical accuracy of the T2D diagnosis estimation by Raman spectroscopy analyses can also be presented using Clarke error grid, which summarizes the performance of the new models for glucose quantitation [66, 67]. Clarke error grid is divided into five zones; values in zones A and B represent accurate or acceptable results; zone C could lead to a bad diagnostic; zone D represents a dangerous fault to detect or treat; and zone E means wrong treatment.

## Results

### In vivo spectra results

**Fig. 1:**
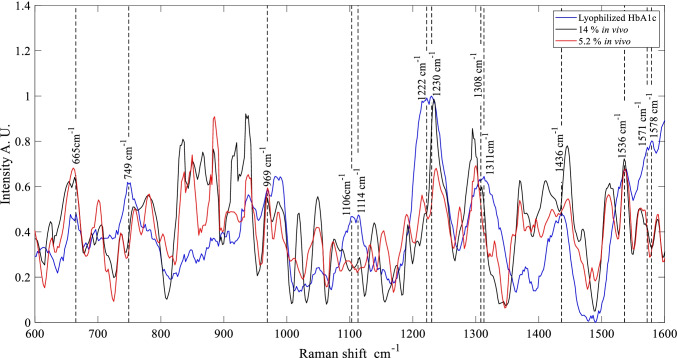
Raman spectra of lyophilized human HbA1c compared with the highest and lowest percentage of HbA1c in vivo measurements in the region of the wrist and their respective peaks

The acquisition of Raman spectra was performed in three different body parts (forearm, wrist, and index finger). The three body parts spectra were compared with Raman measures of lyophilized glucose and lyophilized HbA1c previously reported [38, 41]. Figure 1 depicts a comparison of the lyophilized HbA1c spectrum with the in vivo spectra from individuals’ wrists, as an example, the highest and lowest HbA1c percentages obtained from laboratory tests (14 and 5.2%, respectively) are shown. Figures S3 and S5 in the supplementary materials show the forearm and finger spectral graphs for the HbA1c comparison. In these graphs, the representative peaks of the lyophilized substances are hightailed by a vertical dotted line; notice that not all the peaks appear in the in vivo measurements; this is due to the different molecular compositions of the skin layers and tissues, among others [68].

We compared and made the peak assignment considering the reported peaks in literature and considering those peaks, which appear not only in the pure substance but also in the individuals’ spectra. For the forearm, the peaks are located at 1536, 1230, 1114, 969, and 665 cm^−1^, for the wrist peasks are located at 1536, 1230, and 665 cm^−1^, and for the index finger at 1536 and 1308 cm^−1^. These peaks correspond to the following molecular vibrations: at 1536 cm^−1^ corresponds Amide II (β-gyre) and a combination of stretching C-N [69, 70], 1230 cm^−1^ corresponds to δ(CmH) [71], 1114 cm^−1^ corresponds to twisting δ(CH2), and stretching C-N in proteins and glucose [70, 72], 969 cm^−1^ corresponds to CH3 deformation, and C-O angel-bending glucose [70, 72], 665 cm^−1^ corresponds to δ(pyr deformation)sym[71, 73], 1308 cm^−1^ corresponds to δasym(CmH) [74].

For the forearm peaks at 544, 837, and 1060 cm^−1^ were analized, for wrist, the peaks at 544 and 837 cm^−1^, index finger at 544 and 837 cm^−1^. Molecular vibrations per each peak are 544 cm^−1^ exocyclic deformation [75], 837 cm^−1^ vibrations ν(C–C) [76], and 1060 cm^−1^ stretching ν(C-O) and ν(C–C) [39, 75].

Signal to noise ratio (SNR) was calculated for each of the 46 volunteers according to Eq. 1, which describes the ratio of highest peak intense mean $\overline{{\varvec{S}} }$ and the standard deviation at this frequency *σ*_y_ [30].

1 $$SNR=\frac{\overline{S}}{{{\varvec{\sigma}} }_{{\varvec{y}}}}$$

The SNR was computed using peaks at 1230 cm^**−**1^ for HbA1c and 1106 cm^**−**1^ for glucose in each of the nine measures acquired per volunteer. The obtained results showed an SNR for HbA1c of 9.75 ± 7.26, 11.63 ± 9.65, and 17.06 ± 29.77 in the forearm, wrist, and index finger, respectively. SNR results for glucose were 4.52 ± 1.70, 5.02 ± 2.34, and 8.28 ± 13.59 in the forearm, wrist, and index finger, respectively. The SNR of glucose is lower than the HbA1c. However, an SNR ≥ 3 was obtained; therefore, it is possible to carry out quantitative analyses [30].

### Non-linear regression based on artificial neural networks

**Fig. 2:**
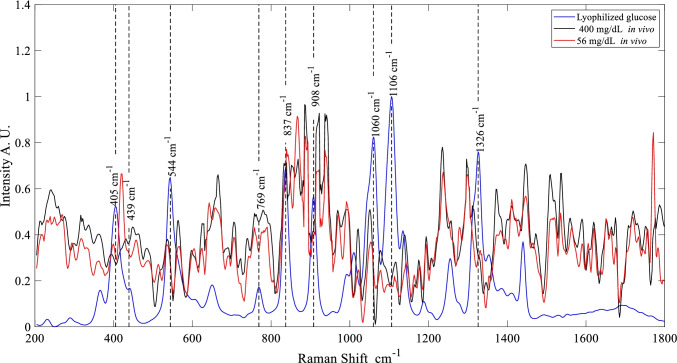
Raman spectra of lyophilized glucose compared with the highest and lowest value of glucose in vivo measurements in the region of the wrist and their respective peaks

Non-linear regression was performed to predict HbA1c and glucose values as accurately as possible in non-invasive measurements using the Raman spectra obtained from the three body parts (Fig. 2). After the signal was pre-processing using Zernike polynomial and Whitaker algorithms to reduce fluorescence and shot noise (see Experimental section), the Raman spectra (200–1800 cm^−1^ and 600–1600 cm^−1^) of the forearm, wrist, and index finger were used as input for the FFNN. The results presented a poor performance for HbA1c and glucose prediction (root mean square error in cross-validation (RMSE-CV) ± standard deviation (SD) = 1.03 ± 0.09%, and 60.32 ± 5.27 mg/dL, respectively) since the main goal is to obtain the lowest RMSE-CV that we can; to improve or at least equal the error in the commercial meters [77, 78], SOM network was implemented in order to improve the previous prediction. Table 1 shows the RMSE-CV and SD for HbA1c and glucose in vivo predictions per body part, in which the value from the laboratory test was considered the ground-truth value. The number of input features to the neural network was equal to the SOM network inputs (788 and 512 features from 200 to 1800 and 600 to 1600 cm^−1^, respectively).

**Table 1:** Regression model performance based on FFNN-SOM to quantify HbA1c and glucose in vivo

Body region	RMSE-CV (HbA1c %) ± SD	RMSE-CV (glucose mg/dL) ± SD
Spectral interval 200–1800 cm^−1^ (788 features)		
Forearm	0.70 ± 0.01	58.43 ± 5.95
Wrist	0.68 ± 0.01	56.42 ± 1.79
Index finger	1.95 ± 0.17	59.25 ± 1.56
Spectral interval 600–1600 cm^−1^ (512 features)		
Forearm	1.41 ± 0.04	56.31 ± 4.28
Wrist	0.85 ± 0.02	58.22 ± 1.03
Index finger	1.90 ± 0.06	56.65 ± 8.99

The best results were obtained from the wrist spectra with RMSE-CV ± SD of 0.68 ± 0.01% and 56.42 ± 1.79 mg/dL, for HbA1c and glucose, respectively, in the interval between 200 and 1800 cm^−1^. For the spectral interval between 600 and 1600 cm^−1^, the wrist region showed the best performance for the HbA1c prediction with 0.85 ± 0.02%; meanwhile, for glucose predictions, the forearm obtained 56.31 ± 4.28 mg/dL (Table 1).

The worst results were obtained using the Raman spectra from the index finger with a RMSE-CV ± SD of 1.95 ± 0.17% and 59.25 ± 1.56 mg/dL in the interval from 200 to 1800 cm^−1^, for HbA1c and glucose, respectively, and 1.90 ± 0.06% and 56.65 ± 8.99 mg/dL for the interval between 600 and 1600 cm^−1^, for HbA1c and glucose, respectively. It is worth pointing out that the width of the wrist and the body mass index (BMI) are not related to the HbA1c percentage according to their correlation coefficient (*R* = 0.0094, 0.073 for width and BMI, respectively), as is shown in graphs reported in the supplementary material (Figs. S1 and S2).

The feature selection method RReliefF combined with the SOM network was implemented in order to improve the predictions of the SOM network alone; the results are shown in Table 2, in which the setting parameters within the best results were obtained. In addition, the RMSE-CV and the SD were calculated via cross-validation in the FFNN for each part of the body. In this case, the interval from 200 to 1800 cm^−1^ was only used, and the best result was obtained in the wrist region using 512 features and 26 k-nearest neighbors for HbA1c predictions with a RMSE-CV ± SD of 0.69 ± 0.07%.

**Table 2:** Regression model performance based on FFNN and RReliefF-SOM for HbA1c and glucose in vivo quantification. The spectral interval between 200 and 1800 cm^−1^ was used to select the mentioned features

	Body region	Forearm	Wrist	Index finger
HbA1c	# Features	100	512	50
	# k-nearest neighbors	13	26	23
	RMSE-CV (HbA1c %) ± SD	1.00 ± 0.08	0.69 ± 0.07	1.52 ± 0.41
Glucose	# Features	200	150	150
	# k-nearest neighbors	9	7	38
	RMSE-CV (glucose mg/dL) ± SD	30.12 ± 0.53	41.44 ± 4.68	49.67 ± 1.66

For glucose predictions, the best result was obtained using 200 features and nine k-nearest neighbors for the Raman spectra from the forearm with a RMSE-CV ± SD of 30.12 ± 0.53 mg/dL. The worst results were obtained using the Raman spectra from the index finger with 1.52 ± 0.41% and 49.67 ± 1.66 mg/dL for HbA1c and glucose predictions, respectively (Table 2).

Of note, the 512 selected features by the RReliefF-SOM method do not necessarily correspond to a specific spectral interval, meaning that these are different points into the whole spectra, without following a specific order; these Raman shifts have been selected by RReliefF due to their capability to predict the response value. Also, it is important to notice that it is not required to test further than 26 k-nearest neighbors since the best results were obtained between 13 and 26 neighbors.

### Clarke error grid results

**Fig. 3:**
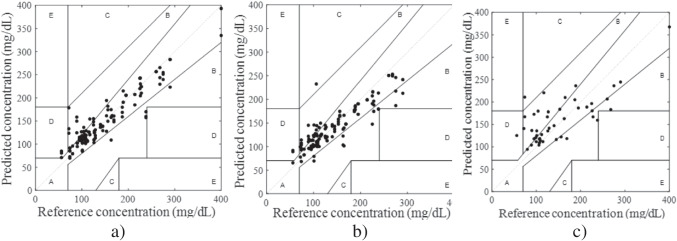
Clarke error grid for the proposed method, **a** forearm, **b** wrist, **c** index finger

Glucose results can be graphically represented using the Clarke error grid, in which a comparison between the laboratory test and Raman spectra prediction is presented in order to visualize the clinical accuracy in such predictions. Figure 3 depicts Clarke error grid in the three body parts, considering the best result per body region (using FFNN, RReliefF, and SOM network with 200 features for the forearm and 150 features for wrist and index finger). Each point in the figure represents predicted glucose measurements. For instance, in the forearm (Fig. 3a), almost all the predicted measurements are concentrated in region A; meanwhile, in the index finger case (Fig. 3c), they are scattered throughout regions A, B, and D.

The percentages per zone were the following: glucose prediction for the forearm obtained 82.61%, 15.22%, 0%, 2.17%, and 0% of success for zones A, B, C, D, and E, respectively. Prediction results from the wrist spectra obtained 47.83%, 47.83%, 2.17%, 2.17%, and E 0%, for zones A, B, C, D, and E, respectively. Prediction results from the index finger obtained a 71.74%, 26.09%, 0.72%, 1.45%, and 0% of success for zones A, B, C, D, and E, respectively. Using the Clarke error grid to analyze the performance in the glucose quantification, it can be inferred which of the three body parts presented a better efficiency considering that the higher the percentage of success in zone A, the better the proposed method will be. In this case, the best result was obtained in the forearm with an accurate percentage in zone A of 82.61%. Although the RMSE-CV is higher compared to commercial glucometers [77, 78], this approach presents an improvement compared to the state-of-art considering our percentage of success in Clarke error grid zone A.

### Comparison among results by using regressors as SVM, LR, and iPLS

We compared the performance of the FFNN with other methods such as support vector machine (SVM), linear regression, and interval partial least square (iPLS) to differentiate our proposal with techniques that have been reported so far in the state-of-art to predict glucose and HbA1c [16, 17, 42, 45, 60, 79]. The inputs for SVM and linear regression were the same used in the FFNN model. In both cases the number of features were the same in each body part: 100, 512, and 50 features for the forearm, wrist, and index finger, respectively, for HbA1c prediction using the SOM network and RReliefF. Meanwhile 200, 150, and 150 features for the forearm, wrist, and index finger, respectively, for glucose prediction. Table 3 presents the results obtained from SVM and linear regression analyses of the measurements performed in the three different body parts. The performance of the FFNN was better than SVM and linear regression for prediction of HbA1c and glucose (0.69, 1.40, 1.76% for HbA1c, respectively; and 30.12, 30.50, 30.56 mg/dL for glucose, respectively).

**Table 3:** Regression model performance based on SVM, and linear regression combined with RReliefF-SOM for HbA1c and glucose in vivo quantification. The spectral interval between 200 and 1800 cm^−1^ was used to select the mentioned features

Support vector machine				
	Body region	Forearm	Wrist	Index finger
HbA1c	# Features	100	512	50
	# k-nearest neighbors	13	26	23
	RMSE-CV (HbA1c % ± SD)	1.53 ± 0.12	1.40 ± 0.17	1.58 ± 0.12
Glucose	# Features	200	150	150
	# k-nearest neighbors	9	7	38
	RMSE-CV (glucose mg/dL ± SD)	30.50 ± 3. 73	45.54 ± 7.25	54.90 ± 8.6
Linear regression				
	Body region	Forearm	Wrist	Index finger
HbA1c	# Features	100	512	50
	# k-nearest neighbors	13	26	23
	RMSE-CV (HbA1c % ± SD)	1.76 ± 0.28	2.34 ± 1.69	2.17 ± 1.12
Glucose	# Features	200	150	150
	# k-nearest neighbors	9	7	38
	RMSE-CV (glucose mg/dL) ± SD	30.56 ± 3.86	79.63 ± 7.96	59.54 ± 9.67

Besides, the FFNN and iPLS performance were compared. These methods were tested within ten intervals of the Raman spectra (200–1800 cm^−1^). Table 4 shows the intervals that provided the best results from the ten spectral intervals, being the interval from 771 to 975 cm^−1^, which provides the best result for HbA1c and glucose prediction using iPLS. In general, FFNN performed better than iPLS. The RMSE-CV of FFNN was 0.69% and 30.12 mg/dL for HbA1c and glucose, respectively; meanwhile, the error of iPLS was 1.85% and 64.55 mg/dL, respectively.

**Table 4:** Regression model performance based on iPLS and FFNN to HbA1c and glucose in vivo quantification. The spectral interval between 200 and 1800 cm^−1^ was used to select the mentioned intervals

	Body region	Forearm	Wrist	Index finger
HbA1c	Spectral interval (cm^−1^)	771—935	771—935	407—593
	iPLS RMSE-CV (HbA1c %)	1.85 ± 0.01	1.94 ± 0.02	1.96 ± 0.03
	FFNN RMSE-CV (HbA1c %)	2.22 ± 0.24	2.10 ± 0.10	1.75 ± 0.08
Glucose	Spectral interval (cm^−1^)	1390–1529	771–935	937–1092
	iPLS RMSE-CV (glucose mg/dL)	65.73 ± 0.32	64.55 ± 0.70	67.42 ± 0.34
	FFNN RMSE-CV (glucose mg/dL)	73.01 ± 7.45	73.73 ± 3.63	50.00 ± 6.25

### Classification based on the HbA1c values

A classification using FFNN was performed, based on the HbA1c percentages from conventional laboratory methods, and three classes were created (healthy, prediabetes, and T2D). The accuracy results of the spectral intervals (200–1800 cm^**−**1^ and 600–1600 cm^**−**1^) are shown in Table 5 without using neither the SOM network nor RReliefF; the best results obtained a classification accuracy of 96.01% in the wrist region using the spectral interval between 600 and 1600 cm^**−**1^ (512 features). It should be noted that the experimental results after using the SOM network present low accuracy (85.60% the highest one, see Table S5 in supplementary materials). Therefore, we do not consider it necessary to implement RReliefF to improve the results.

**Table 5:** Classification model performance per body region

Spectral intervals	Forearm (% accuracy) ± SD	Wrist (% accuracy) ± SD	Index finger (% accuracy) ± SD
200–1800 cm^−1^ (788 features)	91.98 ± 5.19	94.75 ± 2.52	80.36 ± 9.18
600–1600 cm^−1^ (512 features)	92.58 ± 4.15	*96.01* ± *2.17*	81.59 ± 9.79

Furthermore, specificity and sensitivity were calculated for the best performing spectral interval of each body region, being this 600–1600 cm^−1^ for the forearm, wrist, and finger index (Table 6); the best sensitivity and specificity percentages were obtained from the Raman spectra of the wrist, being 90.00% and 99.73%, respectively; and the worst results were obtained from the index finger Raman spectra. In addition, the specificity and sensitivity of the predicted values of HbA1c obtained through Raman spectroscopy and FFNN were 60.71% and 93.10%, respectively (Table 7).

**Table 6:** Sensitivity and specificity per body region using FFNN and three classes

Body region	Metric	Healthy	Prediabetes	T2D
Forearm	Sensitivity	83.33%	87.78%	98.26%
	Specificity	99.18%	97.51%	90.83%
*Wrist*	*Sensitivity*	*94.44%*	*90.00%*	*98.61%*
	*Specificity*	*99.73%*	*98.45%*	*92.74%*
Index finger	Sensitivity	25.00%	70.00%	95.83%
	Specificity	98.26%	95.96%	60.00%

**Table 7:** Sensitivity and specificity obtained from the regression based on FFNN and Raman spectroscopy in the wrist region

Metric	Healthy	Prediabetes	T2D
Sensitivity	100.00%	60.71%	87.50%
Specificity	93.10%	87.96%	80.77%

## Discussion

Currently, the global burden associated with T2D is estimated to be 67.9 million disability-adjusted life-years (DALYs); the latest projections point towards an increment of 11.4, resulting in 79.3 million by 2025 [80]. Furthermore, underdiagnosis of T2D remains as a key problem in low- and middle-income countries [5]. It is proposed that the development of low-cost and non-invasive methods could alleviate this problem. The development of non-invasive methods could potentially alleviate the increasing environmental footprint of health care associated to diagnostic methods that requires many contaminants, and single-use tests [81, 82]. Here, we showed that the implementation of FFNN for the analysis of the non-invasive quantification of HbA1c and glucose by means of Raman spectroscopy enhances its ability to identify subjects with T2D.

The Raman spectra in vivo measurements have been used for the determination of T2D diagnosis biomarkers in order to discriminate among T2D and healthy patients [16, 17]. Also, non-invasive measurements to quantify glucose have used different regression methods [42, 46]. In this work, the Raman spectra from three different parts of the body in 46 individuals were acquired, from which a spectral analysis was made to identify the representative peaks of glucose and HbA1c, since they have been reported in blood samples [15, 83], lyophilized HbA1c, and different concentrations [79], even in vivo measurements [16, 17].

Moreover, FFNN was implemented to quantify HbA1c and glucose concentrations using the Raman spectra. However, feature selection methods and SOM networks were required to improve the results due to the low intensity and noisy signal in the Raman spectra (supplementary material, Tables S1–S4). Remarkably, the Hba1c and glucose Raman spectra obtained in the wrist and forearm performed better than the fingertip. This could be explained by the collagen-enriched tissue in the fingertips. Given the fact that the collagen has its own Raman spectra [84], these spectra may interfere in the quantification of glucose and Hba1c. In addition, the epidermis layer is thicker than the forearm and wrist and it might have a high variation among individuals [85, 86].

In order to overcome the above-mentioned technical limitations of the spectroscopy, we proposed the use of FFNN. It is important to notice that the FFNN can approximate any fitting of a data set representing a relationship [87]. Considering this, FFNN was implemented to Raman spectra without neither data selection nor enhancing data; however, the in vivo Raman spectra are noisy data, and the prediction error was high (1.03 ± 0.09%, and 60.32 ± 5.27 mg/dL for HbA1c y glucose, respectively). In order to reduce the error in the predictions, an early selection of valuable features should be conducted. Therefore, we decided to use feature selection and extraction methods, such as the SOM network and RReliefF, which have not been implemented in Raman spectra.

SOM is an unsupervised classification algorithm [57] that allows us to know the distribution of the data in function of intrinsic features of the data and generate prototypes based on the HbA1c and glucose concentrations, which helps us to have a better representation of the signal (reducing the RMSE-CV). Despite that, this representation has the same number of features as the input signal has. It should be noted that the fewer features the problem has, the shorter the FFNN execution time; this is suitable for an immediate result and a future embedded application. For this reason, we use feature selection methods, as with RReliefF, among others (see supplementary material), and the number of features that obtained the best result per each part of the body is presented in Table 2.

Besides, a comparison between FFNN, SVM, and LR was made using the best-case per body region. The used features for HbA1c were SOM network in the spectral interval from 200 to 1800 cm^−1^ for the forearm, and 512 selected features by RReliefF-SOM for the wrist. It is worth mentioning that these characteristics do not correspond to the spectral region from 600 to 1600 cm^−1^. And for the index finger, 50 features were selected by RReliefF-SOM. For the glucose case, the used features were 200, 150, and 150 using RReliefF and SOM for the forearm, wrist, and index finger, respectively.

Results shown in Table 3 depict that the error obtained using SVM and LR is higher than that achieved by the FFNN (0.69% ± 0.07%), and comparing SVM and LR, the first one has better performance; this may be since different kernels were used to perform the regression, that means, kernels are not necessarily linear. Thereby, linear regressions may not present a good performance in HbA1c quantification. In the glucose case, the results from the forearm region for LR and SVM were very close to the FFNN results. However, our proposal is still better with RMSE-CV and a standard deviation of 30.12 ± 0.53 mg/dL (see Table 2).

Former studies in which Raman spectroscopy had been used to estimate the glucose values have evaluated the specificity and sensitivity in relation to the binary classification (healthy individuals vs. controlled T2D), reporting both specificity and sensitivity of 100% [16]. In our case, these parameters were calculated in multiclass classification, and the best results were 94.44% sensitivity and 99.73% specificity in the healthy class. On the other hand, previous studies had used FFNN to classify subjects in two categories (healthy and T2D) [17]. Their best result was 96% of accuracy and sensitivity and specificity values of 88.9% and 90.9%, respectively. An accuracy of 96.01% was similarly obtained implementing our methodology, despite the difference is not significant (*P* > 0.05, *P* = 0.31 using the non-parametric method Kruskal Wallis); an improvement is observed in the percentages of sensitivity and specificity; although it should be considered that this study [17] was obtained through a binary classification, while the present work is a multiclass classification. Hence, our proposed methodology outperforms the already published state-of-the-art methods.

Sensitivity and specificity were calculated from the predicted HbA1c percentages by the FFNN. The results are shown in Table 7, in which the percentage metrics were 100–93.10% for healthy, 60.71–87.97% for prediabetes, and 87.50–80.70% for T2D, which means a decrease for prediabetes and T2D groups; this is due to the error obtained in the regression model. Concerning the works reported in the literature [76, 88], there have been reported sensitivity and specificity for the A1c commercial test (boronated affinity high-performance liquid chromatography-HPLC) with values for prediabetes in a range of 84 to 95% and 86 to 93%, respectively, and for T2D at around 45 and 99% [72] and also 44 and 79%, respectively [88]. That means that even invasive commercial tests are imperfect and may present low sensitivity. Moreover, this is the first approximation to the development of a painless method since no work has been reported nowadays in order to obtain an in vivo quantification of HbA1c by non-invasive techniques such as Raman spectroscopy.

Although several investigations have been made in order to achieve reliable quantification of HbA1c, a combination like the one presented in this work had not been reported so far, which consists of different concentrations in a population (46 individuals) of multiple individuals with 36 different concentrations of HbA1c and 43 concentrations of glucose, as well as the combination of feature selection methods and artificial neural networks. Our proposal obtained a RMSE-CV carried out from the Raman measurements taken on the wrist in a range of 5.2–14% of HbA1c of 0.69%. This is the first work implementing non-invasive measures to quantify HbA1c in humans.

Regarding the glucose measurements, we obtained a RMSE-CV and standard deviation of 30.12 ± 0.53 mg/dL in the forearm region, and in the percentage of success in Clarke error grid for zone A 82.61%, zone B 15.22%, zone D 2.17%, and zones C and E 0%, the glucose values varied from 56 to 400 mg/dL. Concerning the reports in the state-of-art, percentages in Clarke error grid have been reported with the following results: 78.4% of success in zone A using partial least square (PLS) from the forearm of 111 individuals [44]; 72% in zone A measured to the middle finger of 29 individuals and applied linear regression [42]; 93% zones A and B by an intra-subject analysis into 35 individuals and implemented critical-depth Raman spectroscopy and PLS [45]; another work reports no incidences in zone D; however, percentages were not reported, and their study was intra-subject [43].

## Conclusion

We showed for first time that Raman spectroscopy could be used to determine the percentage of HbA1c in vivo. In addition, we improved the success in zone A of Clarke error grid for the glucose quantification, which represents accurate or acceptable results of alternative glucose measurement in vivo without blood extraction. Therefore, using Raman spectroscopy combined with feature selection methods and artificial neural networks provided the first step for a non-invasive and environmentally responsible approach to measuring glucose and HbA1c.

## Supplementary Information

Below is the link to the electronic supplementary material.

Supplementary file1 (DOCX 497 KB)

## Data Availability

The data that support the findings of this study are openly available in the Center for Open Science repository at https://doi.org/10.17605/OSF.IO/V32D4 and the medical protocol is available upon reasonable request.
