# Supplementary material for: Quantification of glycated hemoglobin and glucose in vivo using Raman spectroscopy and artificial neural networks
Source: Lasers Med Sci. 2022 Sep 5;37(9):3537–49. doi: 10.1007/s10103-022-03633-w (PMC9708775; doi:10.1007/s10103-022-03633-w)
Supplement: Supplementary file 1 — Supplementary file1 (DOCX 497 KB) [file 10103_2022_3633_MOESM1_ESM.docx]

Supporting Information

Title: Quantification of glycated hemoglobin and glucose in vivo using Raman spectroscopy and Artificial Neural Networks.

N. González-Viveros^1^ | J. Castro-Ramos^1^ | P. Gómez-Gil^2^ | H. H. Cerecedo-Núñez^3^ | F. Gutiérrez-Delgado^4^ | E. Torres-Rasgado^5^ | R. Pérez-Fuentes^6^ | J. L. Flores-Guerrero^7, *^

^1^ Optics coordination, National Institute of Astrophysics, Optics and Electronics (INAOE), 72840, Puebla, México

^2^ Computer Science coordination, National Institute of Astrophysics, Optics and Electronics (INAOE), 72840, Puebla, México.

^3^ Faculty of Physics, Veracruzan University (UV), 91090, Veracruz, México.

^4^ Centro de Estudios y Prevención del Cáncer, (CEPREC), 29038, Chiapas, México.

^5^ Faculty of Medicine, Meritorious Autonomous University of Puebla (BUAP), 72589, Puebla, México.

^6^ Department of Chronic Disease Physiopathology, East Center of Biomedical Research, Mexican Social Security Institute (CIBIOR), 74360, Puebla, México.

^7^ MRC Unit for Lifelong Health and Ageing, Institute of Cardiovascular Science, University College London, WC1E 7HB, London, United Kingdom.

*Correspondence

J. L. Flores-Guerrero, MRC Unit for Lifelong Health and Ageing, Institute of Cardiovascular Science, University College London, WC1E 7HB, London, United Kingdom.

Email: j.flores-guerrero@ucl.ac.uk

**Additional Methodological Information**

We acquired the Raman spectra from three different parts of the body in 46 individuals, we implement a correlation to identify the relation between the HbA1c measurement through Raman spectroscopy, the standard laboratory technique, the wrist circumference, and Body Mass Index (BMI). Figures S1 and S2 show the results.

In addition, we made a spectral analysis to identify the representative peaks of glucose and HbA1c. Figures S3 to S6 shows a comparison of the lyophilized glucose and HbA1c spectrum with the in vivo spectra from individuals’ forearm and index finger; as an example, the highest and lowest values of glucose and HbA1c obtained from laboratory tests are shown (400 and 56 mg/dL, and 14 and 5.2%, respectively).

To obtain the minimum error in predicting HbA1c and glucose, we implemented feature selection and extraction methods. The compared methods were Correlation Feature Selection (CFS) [1], the Wrapper method [2], both implemented in the free software WEKA from the Waikato University in New Zealand [3], and Principal Component Analysis (PCA) [4] implemented in MATLAB 2019b, for the results see tables S1, S3, and S4. As input to the FFNN used the selected features and implemented the three cross-fold validation. We report the Root Mean Square Error in Cross-Validation (RMSE-CV) and the standard deviation between folds.

In addition, the intensity of the representative peaks was compared per concentration in volunteers' spectra. For glucose, figure S7 shows a comparison between the peaks intensity and concentration in volunteers’ forearm (this zone obtained the lowest error in the regression results). Peaks at 1125 and 1366 cm^-1^ presented an upward trend as the concentration increased, this can be visualized by implementing a trend line as it is shown for the peak at 1125 cm^-1^. The analysis of HbA1c is shown in figure S8, the wrist was the analyzed zone due to it presented the best result in the regression model. Peaks at 1114, 1230, 1308, 1436, and 1571 cm^-1^ presented an upward pattern as the concentration increased, the trend line for 1571 cm^-1^ is shown.

PCA was applied to the obtained results of the SOM network. Table S2 presents the outcomes, in which SOM improved the results in the PCA analysis. 100 PCs were selected with a cumulative variance upper than 90%. Table 3 shows the results of CFS implementation; this algorithm used three folds, and the ANN used the features that appeared at least in one-fold. The wrapper method used three folds and the perceptron multilayer as an induction training algorithm. Table S4 shows the results.

We computed multiclass classification based on HbA1c values to individuals. It applied the SOM network to enhance the signal as in the regression model; however, the results in Table S5 show a decrease in the accuracy percentage (96.01% in the wrist region without using neither the SOM network nor RReliefF).


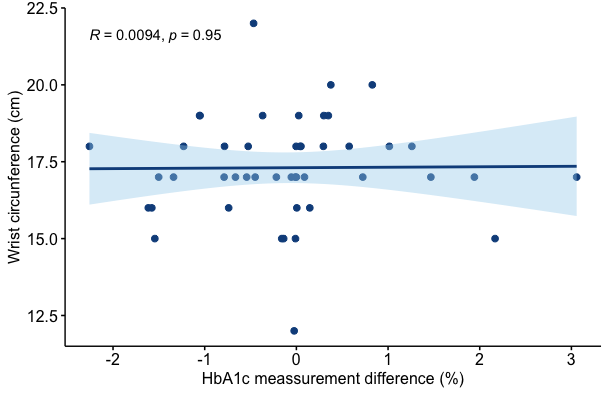


Figure S1 Differences between the HbA1c measurement by means of Raman spectroscopy and standard laboratory technique are not associated with the wrist circumference.


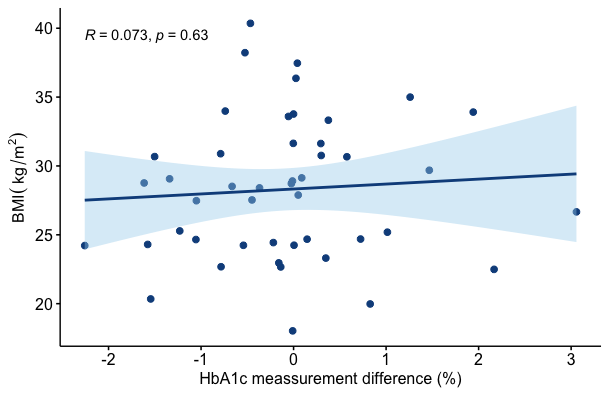


Figure S2 Differences between the HbA1c measurement by means of Raman spectroscopy and standard laboratory technique are not associated with the body mass index (BMI).


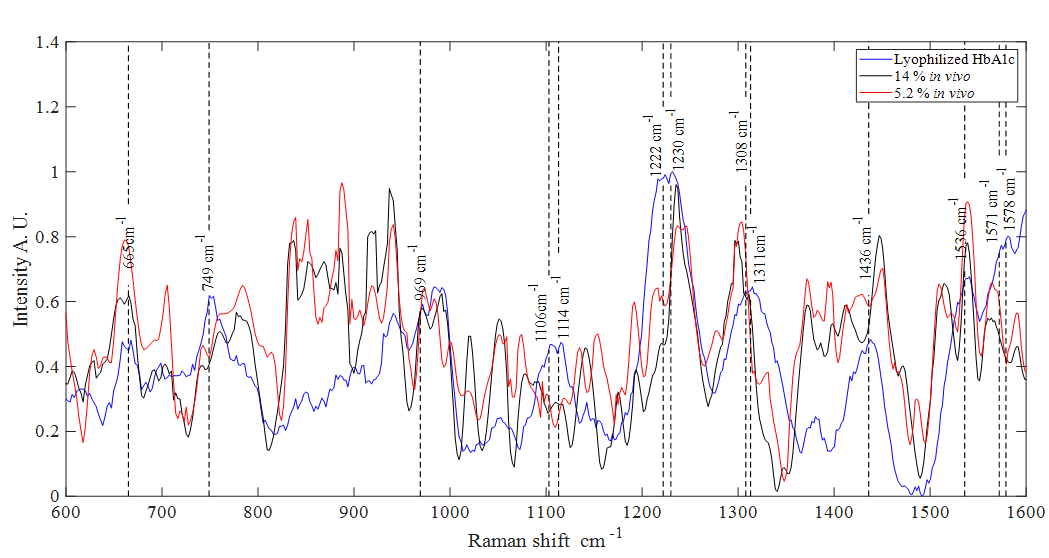


Figure S3 Raman spectra of lyophilized human HbA1c compared with the highest and lowest percentage of HbA1c in vivo measurements in the region of the forearm and their respective peaks.


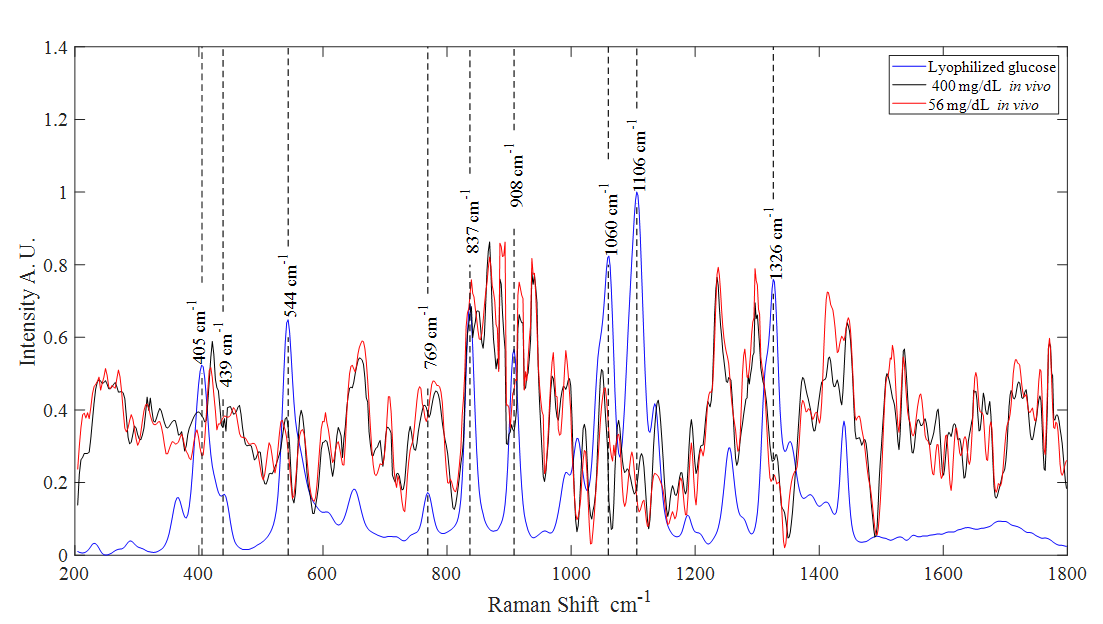


Figure S4 Raman spectra of lyophilized glucose compared with the highest and lowest value of glucose in vivo measurements in the region of the forearm and their respective peaks.


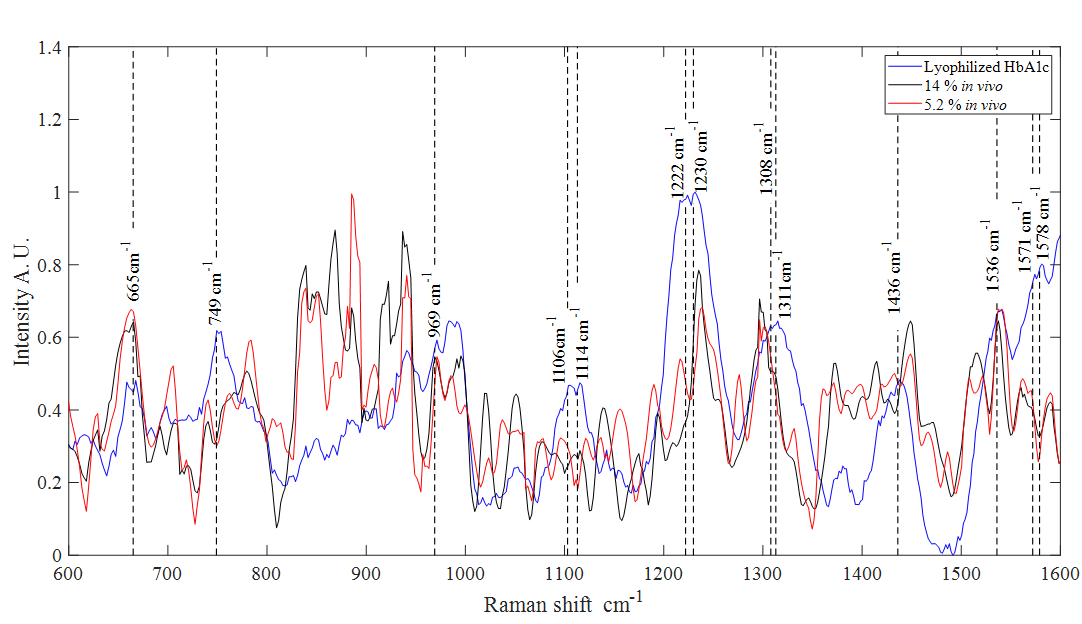


Figure S5 Raman spectra of lyophilized human HbA1c compared with the highest and lowest percentage of HbA1c in vivo measurements in the region of the index finger and their respective peaks.


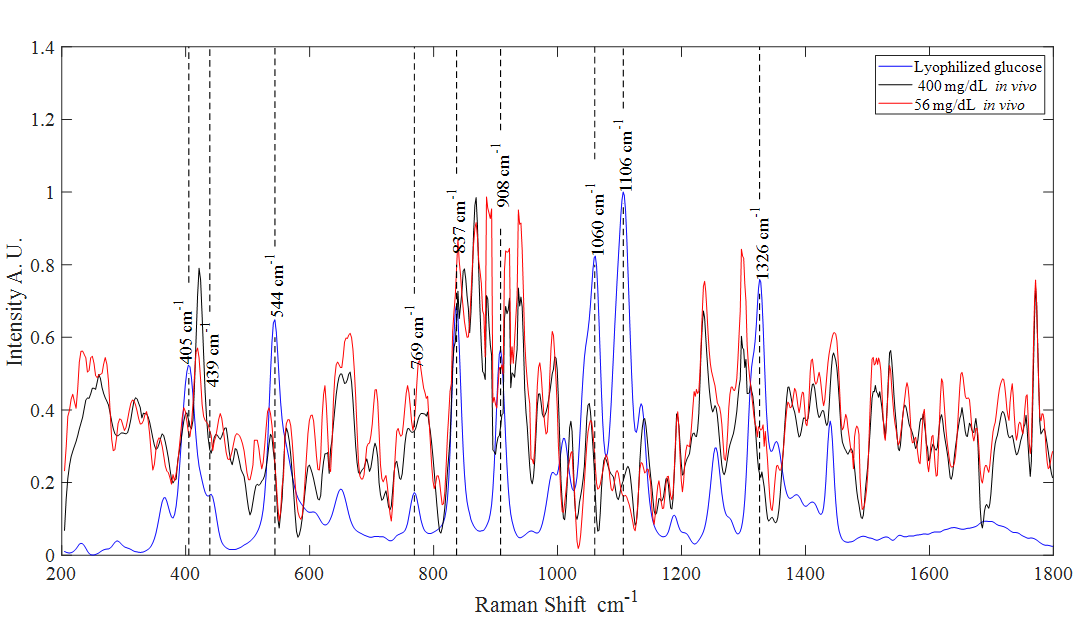


Figure S6 Raman spectra of lyophilized glucose compared with the highest and lowest value of glucose in vivo measurements in the region of the index finger and their respective peaks.

Figure S7 Intensity vs concentration in representative peaks of glucose in the volunteers' forearm.

Figure S8 Intensity vs concentration in representative peaks of HbA1c in the volunteers’ wrist.

Table S1 Regression model performance of the FFNN and PCA implementation, the first 100 principal components were used for each spectral interval.

| **Spectral interval 200 – 1800 cm^-1^** | | |
| --- | --- | --- |
| **Body Region** | **HbA1c RMSE-CV (%)** | **Glucose RMSE-CV (mg/dL)** |
| **Forearm** | 2.30 ± 0.26 | 73.91 ± 5.35 |
| **Wrist** | 2.25 ± 0.16 | 74.66 ± 5.46 |
| **Index finger** | 1.65 ± 0.14 | 51.50 ± 3.60 |
| **Spectral interval 600 – 1600 cm^-1^** | | |
| **Forearm** | 2.29 ± 0.52 | 73.22 ± 6.96 |
| **Wrist** | 2.09 ± 0.16 | 76.50 ± 4.93 |
| **Index finger** | 1.70 ± 0.06 | 47.15 ± 0.89 |

Table S2 Regression model performance of the FFNN, PCA, and SOM implementation, the first 100 principal components were used for each spectral interval.

| **Spectral interval 200 – 1800 cm^-1^** | | |
| --- | --- | --- |
| **Body Region** | **HbA1c RMSE-CV (%)** | **Glucose RMSE-CV (mg/dL)** |
| **Forearm** | 1.71 ± 0.14 | 62.24 ± 8.9 |
| **Wrist** | 1.78 ± 0.14 | 63.97 ± 2.37 |
| **Index finger** | 1.81 ± 0.10 | 38.23 ± 4.45 |
| **Spectral interval 600 – 1600 cm^-1^** | | |
| **Forearm** | 1.73 ± 0.15 | 67.77 ± 10.28 |
| **Wrist** | 1.86 ± 0.12 | 63.51 ± 6.11 |
| **Index finger** | 1.86 ± 0.13 | 39.39 ± 5.99 |

Table S3 Regression model performance of the CFS and SOM implementation, the spectral interval between 200 and 1800 cm-1 was used to select the features.

| **Search Algorithm “BestFirst”** | | | | |
| --- | --- | --- | --- | --- |
|  | **HbA1c** | | **Glucose** | |
| **Body region** | **# Features** | **RMSE-CV (%)** | **# Features** | **RMSE-CV (mg/dL)** |
| **Forearm** | 27 | 1.60 ± 0.05 | 31 | 46.23 ± 2.76 |
| **Wrist** | 28 | 1.52 ± 0.07 | 21 | 55.44 ± 2.21 |
| **Index finger** | 28 | 2.07 ± 0.24 | 21 | 67.65 ± 7.76 |
| **Search Algorithm “Greedy Stepwise”** | | | | |
| **Body region** | **# Features** | **RMSE-CV (%)** | **# Features** | **RMSE-CV (mg/dL)** |
| **Forearm** | 21 | 1.66 ± 0.10 | 21 | 48.72 ± 5.39 |
| **Wrist** | 24 | 1.56 ± 0.09 | 13 | 57.60 ± 1.94 |
| **Index finger** | 16 | 1.94 ± 0.01 | 17 | 65.82 ± 9.63 |

Table S4 Regression model performance of the Wrapper and SOM implementation, the spectral interval between 200 and 1800 cm-1 was used to select the features.

| **Search Algorithm “BestFirst”** | | | | |
| --- | --- | --- | --- | --- |
|  | **HbA1c** | | **Glucose** | |
| **Body region** | **# Features** | **RMSE-CV (%)** | **# Features** | **RMSE-CV (mg/dL)** |
| **Forearm** | 21 | 1.47 ± 0.01 | 24 | 54.83 ± 6.45 |
| **Wrist** | 24 | 1.52 ± 0.03 | 26 | 53.71 ± 8.59 |
| **Index finger** | 15 | 1.89 ± 0.22 | 26 | 62.42 ± 8.38 |
| **Search Algorithm “Greedy Stepwise”** | | | | |
| **Body region** | **# Features** | **RMSE-CV (%)** | **# Features** | **RMSE-CV (mg/dL)** |
| **Forearm** | 10 | 1.62 ± 0.08 | 6 | 64.72 ± 2.61 |
| **Wrist** | 12 | 1.51 ± 0.05 | 9 | 60.39 ± 2.75 |
| **Index finger** | 13 | 1.94 ± 0.13 | 9 | 67.25 ± 5.14 |

Table S5 Classification model performance using FFNN and the SOM network

| **Spectral intervals** | **Forearm**  **(% accuracy) ± SD** | **Wrist**  **(% accuracy) ± SD** | **Index finger**  **(% accuracy) ± SD** |
| --- | --- | --- | --- |
| **200 - 1800 cm^-1^ (788 Features)** | 84.98 ± 1.54 | 83.14 ± 2.90 | 80.29 ± 9.76 |
| **600 -1600 cm ^-1^ (512 features)** | 83.53 ± 3.31 | *85.60 ± 1.80* | 73.04 ± 5.99 |

Table S6 STARD 2015 EQUATOR List of Essential Items for Reporting Diagnostic Accuracy Studies [5].

| **Section & topic** | **No** | **Item** | **page** |
| --- | --- | --- | --- |
| **Title or abstract** |  |  |  |
|  | 1 | Identification as a study of diagnostic accuracy using at least one measure of accuracy (such as sensitivity, specificity, predictive values, or AUC) | 1 |
| **Abstract** |  |  |  |
|  | 2 | Structured summary of study design, methods, results, and conclusions (for specific guidance, see STARD for Abstracts) | 1 |
| **Introduction** |  |  |  |
|  | 3 | Scientific and clinical background, including the intended use and clinical role of the index test | 1-2 |
|  | 4 | Study objectives and hypotheses | 2 |
| **Methods** |  |  |  |
| **study design** | 5 | Whether data collection was planned before the index test and reference standard were performed (prospective study) or after (retrospective study) | 3 |
| **participants** | 6 | Eligibility criteria | 2 |
|  | 7 | On what basis potentially eligible participants were identified (such as symptoms, results from previous tests, inclusion in the registry) | 2 |
|  | 8 | Where and when potentially eligible participants were identified (setting, location and dates) | 2 |
|  | 9 | Whether participants formed a consecutive, random, or convenience series | 3 |
| **test methods** | 10a | Index test, in sufficient detail to allow replication | 3 |
|  | 10b | Reference standard, in sufficient detail to allow replication | 2 |
|  | 11 | Rationale for choosing the reference standard (if alternatives exist) | NA |
|  | 12a | Definition of and rationale for test positivity cut-offs or result categories of the index test, distinguishing pre-specified from exploratory | 2 |
|  | 12b | Definition of and rationale for test positivity cut-offs or result categories of the reference standard, distinguishing prespecified from exploratory | 2 |
|  | 13a | Whether clinical information and reference standard results were available to the performers/readers of the index test | 1 |
|  | 13b | Whether clinical information and index test results were available  to the assessors of the reference standard | 1 |
| **Analysis** | 14 | Methods for estimating or comparing measures of diagnostic accuracy | 3 |
|  | 15 | How indeterminate index test or reference standard results were handled | NA |
|  | 16 | What missing data on the index test and reference standard were handled | 2 |
|  | 17 | Any analyses of variability in diagnostic accuracy, distinguishing pre-specified from exploratory | NA |
|  | 18 | Intended sample size and how it was determined | NA |
| **Results** |  |  |  |
| **Participants** | 19 | Flow of participants, using a diagram | NA |
|  | 20 | Baseline demographic and clinical characteristics of participants | 2 |
|  | 21a | Distribution of severity of disease in those with the target condition | NA |
|  | 21b | Distribution of alternative diagnoses in those without the target condition | NA |
|  | 22 | Time interval and any clinical interventions between index test and reference standard | 2 |
| **Test results** | 23 | Cross-tabulation of the index test results (or their distribution)  by the results of the reference standard | 3-7 |
|  | 24 | Estimates of diagnostic accuracy and their precision (such as 95% confidence intervals) | 3-7 |
|  | 25 | Any adverse events from performing the index test or the reference standard | 3 |
| **Discussion** |  |  |  |
|  | 26 | Study limitations, including sources of potential bias, statistical uncertainty, and generalizability | 7-8 |
|  | 27 | Implications for practice, including the intended use and clinical role of the index test | 6 |
| **Other information** |  |  |  |
|  | 28 | Registration number and name of registry | 2 |
|  | 29 | Where the full study protocol can be accessed | 1 |
|  | 30 | Sources of funding and other support; the role of funders | 8 |

**REFERENCES**

[1] M. Doshi & S. K. Chaturvedi, International Journal of Computer Networks & Communications (IJCNC) **2014**, 6.

[2] R. Kohavi & G. H. John, Artificial Intelligence **1997**, 97, 273–324.

[3] Weka 3 - Data Mining with Open-Source Machine Learning Software in Java, [cited 2021 November], https://www.cs.waikato.ac.nz/~ml/weka/tips_and_tricks_old.html.

[4] S. Sarkar, U. Taraphder, S. Taraphder, et al., International Journal of Livestock Research **2017**, 7, 60.

[5] Bossuyt, J. B. Reitsma, D. E. Bruns, et al., BMJ **2015**, 351: h5527
